# Clinical Applications of Artificial Intelligence (AI) in Human Cancer: Is It Time to Update the Diagnostic and Predictive Models in Managing Hepatocellular Carcinoma (HCC)?

**DOI:** 10.3390/diagnostics15030252

**Published:** 2025-01-22

**Authors:** Mario Romeo, Marcello Dallio, Carmine Napolitano, Claudio Basile, Fiammetta Di Nardo, Paolo Vaia, Patrizia Iodice, Alessandro Federico

**Affiliations:** 1 Hepatogastroenterology Division, Department of Precision Medicine, University of Campania Luigi Vanvitelli, 80138 Naples, Italy; mario.romeo@unicampania.it (M.R.); carmine.napolitano1@studenti.unicampania.it (C.N.); claudio.basile@studenti.unicampania.it (C.B.); fiammetta.dinardo@studenti.unicampania.it (F.D.N.); paolo.vaia@studenti.unicampania.it (P.V.); alessandro.federico@unicampania.it (A.F.); 2 Oncology Division, Monaldi Hospital, 80131 Naples, Italy; patrizia.iodice@ospedalideicolli.it

**Keywords:** artificial intelligence, hepatocellular carcinoma, precision medicine

## Abstract

In recent years, novel findings have progressively and promisingly supported the potential role of Artificial intelligence (AI) in transforming the management of various neoplasms, including hepatocellular carcinoma (HCC). HCC represents the most common primary liver cancer. Alarmingly, the HCC incidence is dramatically increasing worldwide due to the simultaneous “pandemic” spreading of metabolic dysfunction-associated steatotic liver disease (MASLD). MASLD currently constitutes the leading cause of chronic hepatic damage (steatosis and steatohepatitis), fibrosis, and liver cirrhosis, configuring a scenario where an HCC onset has been reported even in the early disease stage. On the other hand, HCC represents a serious plague, significantly burdening the outcomes of chronic hepatitis B (HBV) and hepatitis C (HCV) virus-infected patients. Despite the recent progress in the management of this cancer, the overall prognosis for advanced-stage HCC patients continues to be poor, suggesting the absolute need to develop personalized healthcare strategies further. In this “cold war”, machine learning techniques and neural networks are emerging as weapons, able to identify the patterns and biomarkers that would have normally escaped human observation. Using advanced algorithms, AI can analyze large volumes of clinical data and medical images (including routinely obtained ultrasound data) with an elevated accuracy, facilitating early diagnosis, improving the performance of predictive models, and supporting the multidisciplinary (oncologist, gastroenterologist, surgeon, radiologist) team in opting for the best “tailored” individual treatment. Additionally, AI can significantly contribute to enhancing the effectiveness of metabolomics–radiomics-based models, promoting the identification of specific HCC-pathogenetic molecules as new targets for realizing novel therapeutic regimens. In the era of precision medicine, integrating AI into routine clinical practice appears as a promising frontier, opening new avenues for liver cancer research and treatment.

## 1. Background

Despite the recent considerable therapeutic progress [1,2], primary hepatobiliary liver tumors (HBLTs) continue to play a significant role in the vast array of cancerous diseases, representing the sixth most common neoplasm for global incidence and ranking third in terms of mortality [3]. Among the HBLTs, the hepatocellular carcinoma (HCC) constitutes the most prevalent histotype, accounting for approximately 80% of all hepatic malignancies [1,3,4].

A multifactorial picture, where various exogenous risk factors promote the onset of this neoplasm in individuals with a susceptible genetic background, depicts the faithful portrait of the complex pathogenesis of HCC [2,4,5,6,7].

Several etiological agents contribute to the chronic hepatitis fueling the progression to advanced fibrosis (AF) and liver cirrhosis stage, where the hazard of HCC dramatically increases [2]; a percentage ranging from 1% to 8% of cirrhotic patients develop HCC annually [2].

Interestingly, different leading etiological factors of the HCC are observed in various geographic areas. The hepatitis B virus (HBV) infection represents the major etiological factor in most parts of Asia (except Japan) [8,9], South America, and Africa [10]; The hepatitis C virus (HCV) infection is the predominant cause in Western Europe, North America, and Japan; and alcohol intake constitutes the leading factor in Central and Eastern Europe [11].

Even though viral-related (HBV and HCV) chronic liver diseases continue to represent a major global factor contributing to the HCC onset, in recent years, the growing prevalence of metabolic dysfunction-associated steatotic liver disease (MASLD), due to the spreading of unhealthy lifestyle habits [12,13,14], has promoted a noticeable shift in the etiological panorama of HCC in Western countries [15,16]. Additionally, the long-term effects of HBV vaccination in newborns, as well as the current availability of effective treatments for viral chronic liver diseases, have substantially contributed to this epidemic-etiological change, simultaneously with the increasing rise of MASLD [2,15]. MASLD currently represents the predominant hepatopathy, rapidly affirming itself as the most common cause of hepatic damage, liver cirrhosis, hepatic transplantation, and HCC [17,18].

Epidemiological projections for HCC in the MASLD context appear even more alarming, considering that obesity and diabetes, which represent crucial cardiometabolic factors configuring the “metabolic dysfunction”, are well-recognized and independent risk factors for HCC [16,19,20,21], in addition to the reported occurrence of this neoplasm even in patients without AF or cirrhosis escaping the dedicated traditional HCC surveillance programs [22,23,24].

Altogether, this evidence stresses the urgent need for a tailored approach, based on the development of tools appropriately stratifying the HCC risk and aiming to implement the most suitable management strategy [25]. In this sense, the identification of HCC in the early stages appears crucial in opening different “resolutive” therapeutic scenarios, that would otherwise be precluded in the more advanced stages of the disease [26]. Nonetheless, the low survival rate, particularly in advanced HCC, combined with the high recurrence rate in these stages, has created a substantial obstacle in the battle against this cancer, reaching what seems to be a plateau in terms of management progress [27].

Therefore, the need to explore new effective approaches appears to be a crucial challenge that has substantially contributed to the adoption of Artificial Intelligence (AI) as a promising ally. In line with this, investigating the potential of AI-based applications for optimizing the identification, diagnostic, and therapeutic strategies for liver cancer represents an urgent need, especially considering the above-presented relative epidemiologic burden and the changes that recently arose in the etiology of this neoplasm.

This review introduces AI and its potential in oncology and aims to explore the current applications of AI in managing HCC across all phases (screening, diagnosis, treatment, and prognosis). In this review, the status of the AI-based approaches for diagnosis and treatment of HCC is presented, providing insights into whether this emerging tool can already be considered a valuable “weapon”, enlarging the available armamentarium against this neoplasm in routine clinical practice.

## 2. Artificial Intelligence: An Emerging Tool Revolutionizing the Management of Human Cancer

AI is defined as the ability of a machine to imitate and replicate cognitive processes typical of human intelligence, such as reasoning, learning, planning, and creativity. AI systems are designed to autonomously adapt their behavior by analyzing the outcomes of previous actions and making independent decisions based on these analyses [28].

In recent years, AI has become a topic of growing interest for the scientific community, with an exponential increase in terms of research, particularly in the oncology field [28,29,30]. AI mimics and implements processes inherent to human intelligence via technological approaches: deep learning (DL) and machine learning (ML).

Machine Learning (ML) focuses on developing algorithms and models that enable systems to learn from data, improving their performance over time without needing explicit programming [31,32]. The primary ML approaches are supervised learning, unsupervised learning, and reinforcement learning. ML, because of its focus on training algorithms to identify patterns and make predictions, holds promise for early disease diagnosis by analyzing large volumes of clinical data [33,34].

A potentially significant role of ML in cancer-related clinical practice has been revealed in radiological imaging. As the most relevant example, the ML application to magnetic resonance imaging (MRI) has been shown to enhance tumor detection in human breast cancer [35]. In particular, the use of both ML-supervised and unsupervised methods for tasks such as anatomical segmentation, lesion identification, texture analysis, and radiomics can contribute to the standardization of image interpretation, thereby improving both sensitivity and specificity, reducing false negatives, and potentially leading to decreased costs and shorter scan durations [35]. Moreover, a recent meta-analysis revealed a sensitivity and specificity comparable to that of a radiologists’ “human reading” in detecting breast cancer in patients undergoing mammography for the “computer-aided detection” (CADe) and “computer-aided diagnosis” (CADx) ML algorithms [36]. However, the retrospective nature of the considered studies, as well as the relatively small sample sizes, represented serious limitations of this research, suggesting the need for large datasets to fully leverage the potential of ML [36]. Additionally, another significant limit of ML application is related to the inherent complexity of the decision-making process, which often complicates the understanding of how a particular response is reached, leading to the characterization of these models as a “black box” [28,29,31].

Deep Learning (DL), utilizes deep neural networks to learn from vast amounts of data [31,32]. A neural network is a computational system inspired by the human brain, designed to detect patterns within the data. These networks consist of nodes, known as “artificial neurons”, which are organized into layers. These layers are categorized into three main types: (a) the first input layer, which receives raw data; (b) middle hidden layers, where complex calculations are performed to extract features and identify patterns within the data; (c) the final output layer, which provides the model’s prediction.

A distinguishing characteristic of DL is the presence of multiple hidden layers in the neural networks, enabling the model to learn progressively more abstract and complex representations of the data [31,32].

In contrast to the traditional ML, DL is particularly adept at handling unstructured data (images and videos), thanks to the power of neural networks [37]. In this sense, DL can be mainly effective in processing complex data, such as medical images (both radiological and histopathological), molecular networks, and genetic sequences, with a large potential range of applications in the oncological field, especially regarding genomics, transcriptomics, and radiomics [38,39,40,41]. Additionally, for the DL approaches utilizing transcriptome data, the utility in predicting primary tumor sites in cancer of unknown origin has been revealed [42,43]. Furthermore, “SCOPE”, a neural network trained on the whole of the transcriptome “The Cancer Genome Atlas Program” (“TCGA”) data, was shown to predict the origins of treatment-resistant metastatic cancers, including rare neoplasms, such as the metastatic adenoid cystic carcinoma [42]. Another relevant research recently presented a DL radio-clinical signature (DLCS) model combining imaging features with clinical risk factors in order to accurately predict the tumor response and to identify the risk of Overall Survival (OS) in locally advanced gastric cancer (LAGC), proposing a novel tool, potentially applicable to guide the personalized treatment approaches through computerized tumor-level characterization [44].

However, similarly to the above-presented ML-related application limits, DL models for image recognition require extensive and well-curated datasets, often consisting of thousands of cases, with the exigence of external validation being a crucial need [28,29,37]. In this sense, assembling such large datasets can be challenging, particularly when the data sources are represented by multiple healthcare centers. Moreover, the variability in the parameters derived from the images produced by scanners from different manufacturers poses another hurdle, as it can affect the consistency and reliability of the extracted features, ultimately complicating the training and generalization of the models across diverse clinical environments [32,37].

Despite these limitations, ML and DL-based approaches promisingly represent encouraging frontiers to be further explored and improved, potentially revolutionizing clinical strategies guiding the management of several human tumors, including HCC.

## 3. Artificial Intelligence in the Management of Hepatocellular Carcinoma

### 3.1. From Standard to Risk-Based HCC Surveillance: AI Contributes to Individual Stratification Risk

Among the several strategies aimed at improving the prognosis of HCC patients, major efforts have been made to characterize an adequate surveillance program, providing early identification of the disease for recognized high-risk individuals, thus ensuring a larger range of “curative”/ “resolutive” therapeutic opportunities [26].

The term “HCC surveillance” is defined as a screening for HCC performed at regular intervals [45]. It is currently estimated that carrying out surveillance has a good cost/benefit ratio; therefore, it is recommended for certain categories of patients.

The standard, not-personalized (according to the tools estimating individual risk) surveillance program, recommended by the European Association for the Study of the Liver (EASL), includes a liver ultrasound (US) performed every six months, which should always be performed by an experienced medical operator [46]. Despite these recommendations, the HCC surveillance program can be optimized and may benefit from improvements, considering that less than half of the European patients who should receive surveillance do not receive it for reasons including lack of awareness, adherence, and access [47]. Additionally, for a considerable portion of patients, the currently available strategy is not effective in properly detecting early HCC, even considering the reported HCC occurrence in patients without AF or cirrhosis in MASLD [22,23], escaping the dedicated traditional HCC surveillance program’s identification.

To optimize the current strategy, emerging evidence has proposed novel, alternative, risk-based surveillance models [48]. In this sense, risk-based surveillance, by tailoring the HCC surveillance strategy to the individual risk of developing HCC, promises to be a superior method compared to the standard approach [48].

The main challenge in this situation is selecting the relatively adequate diagnostic test and stratifying individual HCC risk, as well as exploring the potential contribution of AI.

Current guidelines suggest adopting MRI as a surveillance tool for HCC in selected patients, for whom the US is ineffective [46]. However, the specific indications, imaging sequences, and intervals for MRI surveillance remain unclear.

Altogether, the current knowledge status on this topic highlights the relevance of properly stratifying the patients for tailored HCC risk, rather than merely evaluating the general performance of a single diagnostic test, in a desirable scenario where the mutual binomial “optimal test—individual risk” should drive the strategy.

In this regard, different HCC risk stratification scores, including the Age–Male–ALBI–Platelets Score (aMAP) [49,50,51] and the Toronto HCC Risk Index (THRI), among others, have been proposed [52]. However, despite these encouraging findings, developing and validating regression models remains a complex challenge, especially considering the wide individual fluctuation of the HCC risk over time, and with the nonlinear changes being hard to estimate using the existing tools [53,54].

**Figure 1:**
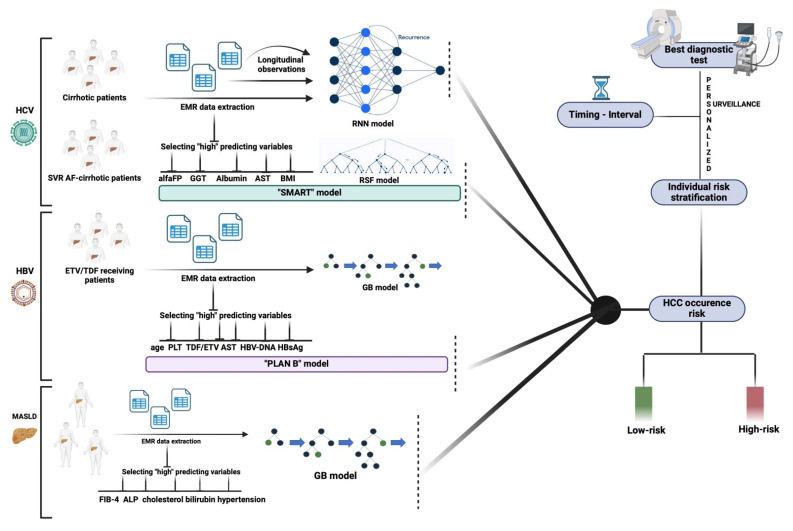
Potential AI contribution to developing tailored HCC surveillance models according to chronic liver disease etiologies. HBV: Hepatitis B virus; HCV: Hepatitis C virus; MASLD: Metabolic dysfunction-associated steatotic liver disease; EMR: Electronic medical record; alfa: alpha-fetoprotein; GGT: gamma-glutamyl transferase; BMI: body mass index; AST: aspartate aminotransferase; RNN: recurrent neural network; RSF: random survival forest; TDF: tenofovir; ETV: entecavir; PLT: platelet count; HBsAg: HBV-surface antigen; FIB-4: Fibrosis 4 score; GB: gradient-boosting.

The rapid expansion of available electronic health record (EHR) longitudinal data has represented a useful aspect of exploiting AI, ensuring some progress both in viral and MASLD settings [25,55,56] (Figure 1).

Ioannou et al. introduced a novel approach for the identification of high-risk patients, which could be particularly useful when the limited resources do not allow for an adequate surveillance of all high-risk patients. The research included 48,151 patients with HCV–related cirrhosis in the national Veterans Health Administration, who had at least 3 years of follow-up after the diagnosis of cirrhosis. The DL recurrent neural network (RNN) models, using raw longitudinal data extracted directly from EHRs, outperformed the conventional linear regression models, suggesting that risk-based HCC outreach and surveillance strategies using RNN models could be used to identify patients with HCV-related cirrhosis with a high risk of developing HCC [56].

In this scenario, individualized HCC surveillance, using risk stratification scores in AF and cirrhotic HCV patients achieving sustained virological response (SVR), represents another relevant question. For this purpose, various risk stratification scores, including the “General Evaluation Score” (GES), have been progressively developed; and moreover, prospective research encouragingly revealed that the use of GES was associated with an improved early-stage detection and the reception of curative treatment [57,58]. Once again, AI appears to ensure a solid contribution even in this research challenge.

Minami et al., in a multicenter cohort study enrolling 1742 HCV-SVR patients, developed an ML-based random survival forest (RSF) model by using seven commonly measured parameters [age, platelet count, alpha-fetoprotein (AFP), gamma-glutamyl transferase (GGT), body mass index (BMI), albumin, and aspartate aminotransferase (AST) (“SMART” model)], showing a good predictive ability and potentially providing a personalized novel surveillance system [59].

For the chronic HBV infection, Kim HY, Lampertico P, et al. aimed to develop and validate an AI-assisted prediction model of HCC risk using a gradient-boosting machine (GBM) algorithm [60]. The research involved a derivation cohort of 6051 chronic HBV-infected patients who received HBV-antiviral therapy (entecavir or tenofovir) and two external validation cohorts. The emerging model, named PLAN-B, comprised ten baseline parameters: cirrhosis, age, platelet count, entecavir/tenofovir, sex, serum ALT and HBV-DNA, albumin and bilirubin levels, and HBeAg status, and it showed significant superiority over the previous models in both validation cohorts [60].

Finally, for the metabolic setting, very recently, Sarkar et al. proposed a predictive model of HCC for MASLD patients, who are becoming the predominant chronic liver patients. A characteristic of MASLD is the occurrence of HCC even in patients without AF, making diagnosis more difficult and less likely to receive a liver transplant. By evaluating a validation cohort of five different ML algorithms, including decision tree (DT), gradient boosted (GB), naïve bayes (NB), probabilistic neural network (PNN), and random forest (RF), the GB-ML model, using EMR data, predicted HCC development at a 92.06% accuracy, with an AUC of 0.97, an F1 score of 0.84, 98.34% specificity, and 74.41% sensitivity [55]. The strongest clinical predictors of HCC in this model were the validated noninvasive tool for liver fibrosis assessment “Fibrosis-4 score” (FIB-4), alkaline phosphatase (ALP), total cholesterol, bilirubin, and hypertension [55].

However, despite these efforts and the encouraging findings in different chronic liver disorders etiologies, the possible exclusive use of AI-based scores to predict the HCC risk remains undefined, mainly because of their dependency on the size and diversity of the training dataset, suggesting that stronger evidence is required to adopt this approach in clinical practice largely.

Therefore, the data supporting the use of other promising modalities (including abbreviated MRI, serum biomarkers, and models) is limited, and the last update of EASL clinical practice guidelines on HCC surveillance continues to recommend a US of the liver every 6 months for HCC screening [61].

### 3.2. AI Facilitates HCC Diagnosis: From Imaging to Histopathology and Transcriptomics

As in a domino, an accurate diagnosis is crucial for adequate grading, staging, and therapeutic approaches, crucially influencing treatment strategies and patient prognosis in HCC. In malignant liver nodules, changes in vascularisation occur and can be appreciated by contrast-enhanced imaging, representing the basis of the non-invasive diagnosis of HCC [45,61].

Relevantly, non-invasive diagnosis can be sufficient when the additional information provided by histopathology will not influence a treatment choice [61].

In some clinical scenarios, histopathology is required, considering the relatively crucial role in confirming the malignant nature of a liver lesion, as well as providing essential insights influencing the therapeutic decision-making (including cellular differentiation, vascular and lymphatic invasion, and metastatic potential) [61].

However, in a routine clinical practice, the approach to a HCC diagnosis recurrently appears nonlinear, making the identification of this neoplasm and the differential diagnosis with other primary or secondary HBLCs a real challenge, particularly in a non-cirrhotic context.

In this scenario, AI-based models may help clinicians to correctly acquire and interpret the obtained results (imaging or histopathologic), facilitating the diagnostic process.

#### 3.2.1. AI Applications Supporting Classical Imaging and Radiomics in Diagnosing HCC

Classical imaging represents the non-invasive standard for HCC definition: in cirrhotic, HBV-infected, or previous HCC-affected patients, a liver nodule can be diagnosed when the major features of HCC are observed with dynamic contrast-enhanced CT, MRI, or contrast enhancement ultrasound (CEUS) following the LI-RADS v2018 recommendations [61]. Routinely, HCC appears chameleonic, and several of the typical major imaging features are faded and not easily identifiable, making the diagnosis of this neoplasm very hard, particularly in patients without a suggestive clinical history (HBV, cirrhosis, etc.). In this sense, radiomics, a technology based on the quantitative extraction of image characteristics from radiological imaging modalities, that permits the identification of crucial imaging biomarkers that cannot be detected visually [62], has made progress in testing the ability of AI frameworks to improve the diagnostic accuracy of US, CT, and MRI in the setting of HCC diagnosis.

*Ultrasound*. The US results represent the first-line imaging modality to identify liver lesions in the clinical workflow. In this context, AI has the potential to assist less experienced radiologists in improving their performance and lowering their dependence on sectional imaging in HCC diagnosis.

Emerging research has investigated the potential role of AI in improving the diagnostic accuracy of the US HCC diagnosis.

In a multicenter study, Yang et al. developed and externally validated a deep convolutional neural network (DCNN) using a large, multicenter database of US images from 13 hospital systems [63].

The resulting model (“DCNN-US”) showed superior diagnostic sensitivity and specificity compared to that of 15-year skilled radiologists and an accuracy comparable to that of a contrast-enhanced CT. The developed model presented an area under the receiver, operating a characteristic curve (AUROC) of 0.92 for differentiating benign from malignant liver lesions. This performance was comparable to (a) the diagnostic accuracy of clinical radiologists (both achieving 76.0%) and (b) the accuracy of contrast-enhanced (CT), showing a diagnostic accuracy of 84.7%, only slightly inferior to MRI at 87.9% [63].

A very recent retrospective study from Thailand incorporated 26,288 US images from 5,444 patients to train the YOLOv5 model, which utilizes DL techniques, for focal liver lesions (FLLs) detection and classification of seven different types of FLLs, including HCC and regenerative nodules. The AI achieved an overall FLLs detection rate of 84.8% (95%CI: 83.3–86.4), with the HCC detection at 82.3% (95% CI: 77.1–87.5), preceded by the cholangiocarcinoma at 92.2% (95%CI: 88.0–96.4) and the focal fatty infiltration FFS at 89.7% (95%CI: 87.1–92.3). The specificities and NPVs for regenerative nodules were 100% and 99.9% (95%CI: 99.8–100.0), respectively [64].

CEUS has been reported to show superior diagnostic performance compared with classical US B-mode images in defining FFLs [65]. For this reason, combining AI with CEUS could be very useful to differentiate benign lesions from HCC or other malignant liver lesions. In 2018, Guo LH et al. proposed a two-stage multiple-view learning framework for CEUS that was a combination of deep canonical correlation analysis and multiple kernel learning (DCCA-MKL), only adopting three typical CEUS images selected from the three phases of the exam (arterial, portal venous and late) [66].

Their results show that this framework best discriminates between benign and malignant liver cancers. Moreover, it was also proved that the three-phase CEUS image-based CAD is feasible for liver cancers with the proposed model [66].

Altogether, the above-presented encouraging findings suggest that AI models can potentially assist physicians in the FLL detection and diagnosis during conventional (CE)US examinations, although further external validation is needed for large-scale clinical application. Table 1 summarizes the principal AI-based models supporting US imaging in diagnosing HCC with the relatively main applications.

*CT and MRI*. CT is routinely used for an accurate diagnosis of HCC, primarily when the results from CEUS are doubtful [61]. In an interesting and highly rated research, to enhance the decision-making of clinicians when diagnosing HCC in cirrhotic patients with indeterminate liver nodules, Mokrane et al. realized a radiomics signature based on 13,920 CT imaging features extracted from data from 178 cirrhotic patients. They used ML techniques for the training, calibration, and validation and reported great accuracy in distinguishing HCC from non-HCC lesions for this signature [67].

More recently, Ho Yu et al. aimed to improve the diagnostic process of HCC diagnosis, particularly targeting the considerable proportions of indeterminate observations recurrently observed in routine clinical practice [68]. The authors developed four DL models for diagnosing HCC on CT via a training-validation-testing approach, identifying, the best-performing model: the Spatio-Temporal 3D Convolution Network (ST3DCN). Relevantly, this model showed better AUCs compared to standard-of-care radiological interpretation [68].

There is a scarcity of research on MRI in HCC due to the technically difficult application of AI in this type of imaging and the costs of manually designing features. A preliminary study by Hamm et al. consisted of the creation of a convolutional neural network (CNN)-based deep learning system (DLS), which demonstrated a 92% accuracy, a 92% sensitivity, and a 98% specificity in classifying liver lesions [69]. Another DL algorithm was created using CNN by *Zhen* et al. to classify liver lesions with MR (enhanced and unenhanced) images and clinical data [70]. CNNs were trained using data from 1210 patients with liver tumours, and models were validated in an external cohort of 201 patients. They also compared the models with the performance of three experienced radiologists. Combining unenhanced images with clinical data greatly improved the performance of the model in classifying HCC with an AUC of 0.985, and the sensitivity and specificity of these models were comparable to that of the three experienced radiologists [70].

Table 2 summarizes the principal AI-based models supporting CT and MRI imaging in diagnosing HCC with the relatively main applications.

#### 3.2.2. AI Applications to Histopathology in Correctly Diagnosing HCC

When the imaging results are inconclusive, histopathology could be crucial in achieving a correct diagnosis of HCC [71,72]. Various efforts are being made to utilize AI to enhance this aspect in order to achieve better disease management, increase the amount of information extracted from a biopsy sample, and potentially remove the bias introduced by manual scoring. The scientific community has focused on relative research on the potential to match or surpass pathologists in specific tasks, even in types of cancer other than HCC [73,74,75].

Limiting the HCC AI applications to human tissue, Lin et al. proposed a fusion between a multiphoton microscopy (MPM) and a DL algorithm for the classification of a HCC differentiation based on a pre-trained VGG-16 framework, reporting an elevated (>90%) accuracy [76].

Subsequently, Chen et al. used histopathological hematoxylin- and eosin-stained slides from the Genomic Data Commons databases to train and externally validate a CNN (“inception V3”) for an automatic classification of this cancer. The authors reported a performance level close to the ability of a 5-year-experienced pathologist, with a 96.0% accuracy for the benign and malignant classifications, and an 89.6% accuracy for good, moderate, and poor tumor differentiation. Additionally, in this study, the capability to predict somatic mutations was evaluated. The model was further trained to identify the ten most common and prognostic mutated genes in HCC, evidencing four of them, including *CTNNB1*, *FMN2*, *TP53*, and *ZFX4*, as predictable from histopathology images, with external the AUCs from 0.71 to 0.89 [77].

Similarly, in further research, aiming to develop and externally validate a CNN-based classification to distinguish HCC from adjacent normal tissues, Liao et al. highlighted the great accuracy and the superiority of a DCNN model compared with the “inception V3” in adopting HCC histopathological slides in order to realise the automatic diagnosis of this cancer, using the features obtained from the TCGA database, simultaneously predicting somatic mutations, thus revealing DL-based histopathology as a promising tool to free pathologists from dull routine practice [78].

More recently, a brilliant study by Kiani et al. aimed to evaluate the effect of DL-based assistance on the diagnostic performance of 11 pathologists, with varying levels of expertise. The model was developed to distinguish the HCC and the cholangiocarcinoma on hematoxylin- and eosin-stained whole-slide images (WSI), achieving accuracies of 0.885 on a validation set of 26 WSI and 0.842 on an independent test set of 80 WSI, respectively.

The model significantly impacted the diagnostic decisions of all pathologists. Furthermore, while the correct predictions contributed to improving the accuracy of the pathologists (Odds Ratio-OR = 4.289), the incorrect predictions reduced it (OR = 0.253), regardless of the experience of the pathologist or the difficulty of the case [79]. Despite the obvious and proven positive sides, the study showcased the potential unintended negative consequences of the use of an AI-based model in the clinical setting.

#### 3.2.3. Besides the Imaging: Exploring the AI Applications to Transcriptomics-Based Approaches

Molecular biology represents yet another promising modern AI-applications field [80,81]. Its contributions to the development of blood tests and the proposal of biomarkers, that are able to identify (even as a screening tool) HCC, would revolutionize the management of this cancer, even in terms of socioeconomic healthcare strategies.

Moreover, Yu et al. identified a panel of fusion genes (created in DNA rearrangements involving the hepatocyte during neoplastic transformation) present in several types of human cancers, including HCC.

Among the eight fusion genes, MAN2A1-FER, TRMT11-GRIK2, and CCNH-C5orf30 were shown as the most frequent in HCC samples, and the relative fusion transcripts in the serum samples of liver cancer patients were identified as circulating, cell-free RNA. Furthermore, the distributions of these gene fusion RNA fragments largely matched those of the primary HCC samples [82].

In more recent research from the University of Pittsburgh, the authors identified the fusion transcripts present in the blood of 61 patients with HCC and 75 patients with liver diseases other than cancer [83]. A computer analysed the data and classified them using ML, generating the hypothesis that four of the fusion transcripts (MAN2A1-FER ≤ 40, CCNH-C5orf30 ≤ 38, SLC45A2-AMACR ≤ 41, and PTEN-NOLC1 ≤ 40) were associated with a high probability of cancer. The researchers then estimated that through the application of this system, it would be possible to correctly predict the presence of the disease in about 83% of cases. A second system, based on the expression of two fusion transcripts (MAN2A1-FER ≤ 40, CCNH-C5orf30 ≤ 38) and serum AFP, showed a predictive capacity approaching 95% [83].

According to the authors, from a bench to bedside point of view, the developed tests may have multiple applications and translational repercussions.

First, these tools could be used as screening tests for the early diagnosis of HCC in patients who have risk factors for this type of tumour. Moreover, these tests could be used to determine whether the patients who discover liver nodules of an unknown nature should undergo a biopsy

Finally, they could be used to evaluate the effectiveness of treatment and the presence of residual disease, taking into consideration the fact that decreased (or zero) levels of fusion transcripts in patients treated for HCC have been observed. Further studies will be needed to validate these hypotheses and the actual reliability of the tests. Still, the simplicity of these methods makes them very interesting and authorises further investigation, even in terms of the AI applications in the prediction of HCC patients’ outcomes.

### 3.3. AI Supports the Development of HCC Predictive Models Influencing Therapeutic Choices

The staging of HCC is essential for the guiding prognosis, treatment decisions, and research, which ultimately contribute to the enhancement of current clinical and epidemiological health practices [27,84].

Several staging systems for HCC have been developed across different regions [10,45,84]. The most widely used prognostic model in Western Europe is the Barcelona Clinic Liver Cancer (BCLC) staging system [26].

This model includes five stages (0, A, B, C, D), based on the extent of the primary lesion, performance status, vascular invasion, and extrahepatic spread [26].

According to this system, the prognosis is primarily influenced by the disease stage at the time of diagnosis and the liver dysfunction severity, impacted either by the tumor itself or by an underlying cirrhosis. For patients diagnosed at the early stages, surgical resection and LT are potentially curative options, with a reported 5-year survival rate of 60–80% [26].

Patients in stage A are considered suitable candidates for a radical surgical resection, while those with stage B or C disease are typically not eligible for resection, but may be candidates for liver transplantation, locoregional therapies such as transarterial chemoembolization (TACE), or systemic treatments [26]. For patients with stage D disease, management should be limited to palliative and supportive care [26].

The importance of implementing a personalized approach for patients with HCC at these stages, aimed at achieving long-term survival outcomes, has driven scientific research toward focusing on the development and training of AI models capable of predicting the likelihood of a treatment response at various stages of the disease.

These AI-driven tools hold significant potential for guiding therapeutic decisions, especially in advanced stages, and improving overall prognosis. Despite the recent, considerable progress [1,2], the HCC continues to represent a major therapeutic challenge, with a dramatically worsening prognosis in stages BCLC-B and C, where the locoregional and systemic treatments, respectively, constitute the predominant choices, impacted by a wide variability in terms of success rate [26,85,86]. Therefore, the potential AI support in predicting the response appears crucial to avoid the related adverse effects, as well as to select the patients who would effectively benefit from that approach.

In a multicenter clinical study, Peng et al. aimed to develop a DL-model for the preoperative prediction of the treatment response in BCLC-B patients receiving TACE [87]. The authors used a total of 789 CT images from three different hospitals and constructed a predictive model leveraging transfer learning techniques with a residual convolutional neural network (ResNet50).

The model’s performance was validated in two independent cohorts. The results demonstrated a high accuracy in predicting the response to TACE therapy, with areas under the curve (AUCs) of 0.97, 0.96, 0.95, and 0.96 for the complete response (CR), partial response (PR), stable disease (SD), and progressive disease (PD), respectively.

More recently, in a multicenter retrospective study, Zeng Q et al. developed an AI model able to estimate the atezolizumab-bevacizumab response signature (ABRS) expression directly from the histological slides and to evaluate whether the model predictions were associated with progression-free survival (PFS) [88]. This study indicates that an AI model applied to HCC digital slides can serve as a biomarker for PFS in patients treated with atezolizumab–bevacizumab, proposing a novel, potentially useful approach to the development of inexpensive and fast biomarkers for targeted therapies [88]. Furthermore, the previously mentioned diagnostic progress related to molecular biology and the combination of AI heatmaps with spatial transcriptomics provides insight into the molecular features associated with predictions, providing an understanding of the biological mechanisms that drive the responses to treatments.

In addition to the disease stage at the time of diagnosis and the related generally poor prognosis in advanced scenarios, the substantial risk of disease recurrence after treatment represents the other relevant variable, severely impacting the prognosis and influencing the HCC mortality rate. In this regard, the possibility of recurrence significantly influences the subsequent treatment decisions and ultimately affects the long-term survival outcomes of patients [89].

A recurrence of HCC is typically categorized as either “early” or “late”, based on the time of diagnosis, with a standard cutoff point of two years [46,61]. A HCC recurrence within 2 years of treatment is defined as “early”, and is generally caused by the occult intrahepatic spread of the primary neoplasm and related to the tumor burden [46,61]. A recurrence occurring after 2 years of treatment is defined as “late”, and is related to de novo HCC, independent of the primary neoplasm. The early HCC recurrence has a significantly poorer prognosis and outcome than the late recurrence [46,61]. In this context, the application of AI to predict the risk of a recurrence in patients treated for HCC could play a crucial role in patient stratification. This would facilitate the implementation of targeted surveillance strategies and guide the decisions regarding the initiation of neoadjuvant or adjuvant therapies following treatment.

Despite the current clinical guidelines recommending surgical resection as the first-line treatment for patients with solitary HCC, the rate of postoperative recurrence remains notably high [26,90]. For this purpose, Ji GW et al. explored the potential of radiomics coupled with ML algorithms in the improvement of the predictive accuracy for the HCC recurrence. In this retrospective study, a total of 470 patients who underwent contrast-enhanced CT and a curative resection for solitary HCC were recruited from 3 independent departments [91]. Using the ML framework, the authors identified a three-feature radiomics signature showing a superior prognostic performance for the prediction of a HCC recurrence, with a C-index of 0.733–0.801, compared to the rival models and widely used staging systems [91]. In another study, Nam JY et al. presented a model for predicting a tumor recurrence after LT by adopting artificial intelligence (“MoRAL-AI”), including 563 patients who underwent LT for HCC at three large LT centers in Korea [92]. The algorithm was developed with a deep neural network (DNN). The largest weighted factors in MoRAL-AI were age, tumor size, age, and serum levels of the AFP and prothrombin time. The authors also proved the advantages of their model in the external validation cohort, with a significantly better discrimination function for predicting a HCC recurrence, compared to other state-of-the-art predictive models, like the Milan criteria [92]. Similarly, Saillard et al. investigated the independent validation sets of two DL algorithms, based on digitized histological slides: the first DL-based algorithm (“SCHMOWDER”) used an attention mechanism on the tumoral areas annotated by a pathologist, whereas the second (“CHOWDER”) worked without human expertise [93]. Relevantly, the c-indices for the survival prediction of SCHMOWDER and CHOWDER reached 0.78 and 0.75, respectively. Both models outperformed a composite score, incorporating all of the baseline variables associated with survival, revealing the vascular spaces, the macro-trabecular architectural pattern, and the lack of immune infiltration as the most predictive values of poor survival in the tumor areas [93].

Similarly, Yamashita et al. developed and validated a DL-based system (“HCC-SurvNet”) to provide risk scores for the disease recurrence after the primary resection, taken directly from hematoxylin- and eosin-stained digital slides of formalin-fixed, paraffin-embedded liver resections [94]. This model stratified the patients into low- and high-risk subgroups, both for OS and PFS (i.e., post-surgical recurrence), reaching concordance indices of 0.724 and 0.683 on the internal and external test cohorts, respectively, exceeding the performance of the standard Tumor-Node-Metastasis classification system [94,95].

More recently, Saito et al. aimed to predict the early recurrence of HCC after the resection, based on digital pathologic images of hematoxylin- and eosin-stained specimens and on ML applying a support vector machine (SVM) [96]. In this research, 158 HCC patients meeting the Milan criteria and receiving a surgical resection were included and further categorized into three groups: Group I, patients with HCC recurrence within 1 year after resection; Group II, patients with HCC recurrence between 1 and 2 years after resection; and Group III, patients with no HCC recurrence within 4 years after resection. Notably, the SVM-based prediction model distinguished the three groups with an elevated (89.9%) accuracy [96]. These findings revealed the relevance of models integrating digital pathology with ML, as a novel potential method for improving the postoperative follow-up, especially for patients at a high risk of early HCC recurrence.

Furthermore, a DL model predicting recurrence-free survival (RFS) on histological slides after LT and resection was developed on a total of 1,118 HCC patients from four independent cohorts, using a nucleus map set (n = 120) to train the U-net on capturing the nuclear architecture [97]. The training set and its nuclear architectural information extracted by the U-net were used to train the MobileNet V2-based classifier (MobileNetV2_HCC_class).

The authors reported the MobileNetV2_HCC_class was a strong predictor of RFS in the LT setting, maintaining a relatively higher discriminatory power than the other factors, even after resection, in the independent validation cohort. Interestingly, a pathological review revealed, among other things, the degree of cytological atypia and nuclear hyperchromasia as the most relevant predictor of histologic features in the areas presenting the highest recurrence [97]. Altogether, the above-presented findings suggest the potential utility of DL- and ML-based approaches on both the imaging data and the histological slides in refining the prognostic prediction of HCC patients and identifying the more adequate individual management after the diagnosis, as well as in the case of recurrence.

## 4. AI Applications in Routine Managing HCC: Are We Ready?

### 4.1. Main Limitations of AI Use in Routine Clinical Practice: Need for Robust Evidence

Despite the recent progress in AI applications for the routine clinical management of human cancer, as for other malignant diseases, even for HCC, a great number of challenges remain, ranging from standardizing and robustly evaluating AI algorithms in prospective studies by using large-scale datasets to establishing the consensus guidelines to ensure accurate and comprehensive reporting of data from the ML and DL research.

As previously shown, the performance of AI algorithms is severely influenced by the amount of data adopted for training. Unfortunately, most of the above-presented studies that were able to consider and enclose a large series of patients were retrospective and thus affected by the relative biases of non-perspective experimental design. In line with this, there is an urgent need to encourage the further deposition of large databases worldwide. In this sense, it is important not to limit this challenge to a restricted geographic area, as to date, the populations considered training the development and training of AI models have lacked sufficient ethnic and socioeconomic diversity. Finally, the heterogeneity of algorithms and software adopted in the currently available research evaluating the application of AI to the clinical management of HCC represents another relevant issue, suggesting that the external validation of all these experimental models is, at the same time, crucial and difficult.

### 4.2. Future Perspectives and Conclusions: How Can the Scientific Community Contribute?

This review of the recent literature highlights the need for further efforts to give AI a concrete supporting role in order for it to be implemented in the clinical practice of HCC management. Although many steps forward have been taken in the last decade, a correct and appropriate application of AI in the routine management of HCC, from diagnosis to treatment, still requires more work [98].

Furthermore, as previously stated, the status of AI applications in the various clinical practice phases appears extremely heterogeneous; while its use currently seems more promising in the areas of diagnosis and prediction of response to therapy, we are still far from its use in the risk prediction and the subsequent stratification of patients for “tailored” screenings.

Prospective studies that irrefutably demonstrate the improvement in the clinical management of patients are still needed, as well as the comparison of new AI models with those already present and used in clinical practice, without underestimating the potential benefits, even in terms of economic and research sources, of adopting transfer learning (TL) approaches [99]. TL represents a type of ML and is an emerging promising technique in which a model trained on one task is reused as the starting point for a new activity [99].

Furthermore, integrating AI-based algorithms into current clinical trials, to authorize the emerging research suggesting their validity as tools for accurately identifying early HCC and predicting therapeutic responses and recurrence, appears to be even more crucial [100,101]. Simultaneously, it is also essential to externally validate any model developed on a real-world population before the clinical implementation, to disavow the presence of any bias highlighted by the trial.

To achieve trials receiving these potential ameliorations, the sharing and deposition of large databases containing imaging, clinical variables, and biological samples, from various departments in different geographic areas, as well as the accessibility of all these data to the scientific community, together with the interpretation and subsequent model-realization performed by AI-dedicated personnel, appears to be crucial [102].

Furthermore, significant benefits can derive from the applications of collaborative-learning-based strategies, consisting of learning approaches involving groups of learners working together to solve a problem, complete a task, or create a product [103].

Moreover, despite the ongoing debate on AI “replacing” humans, the progress shows the increasing need for the pertinent use of these tools in a clinical setting, to insert input data coherently, and to interpret the output data correctly.

The eXplainable Artificial Intelligence (XAI) can significantly contribute to this challenge, representing an encouraging impulse for further progress and a novel frontier in this research field, particularly in reference to interpretations and applications of multiparametric tools [104,105,106]. XAI represents a rapidly emerging branch of ML, aiming to unravel the predictions of complex models, especially those required in healthcare when the diagnoses, recommendations, and treatment choices might rely on the decisions made by AI-based systems [107]. In pioneering research, Lacalamita et al. aimed to develop a supervised learning framework based on a hierarchical community detection and AI models to distinguish the patients and controls, using publicly available microarray data [104]. The authors identified 20 gene communities that discriminated between healthy and cancerous samples with an elevated accuracy, validating the performance even on an independent dataset. Subsequently, after selecting two communities based on relevant biological functions, an XAI-based approach assessed the contribution of single genes to the relative classification task [104].

These findings proposed an effective tool contributing to the recognition of gene communities, helping reveal crucial mechanisms sustaining the HCC onset, and identifying biomarkers potentially useful in a clinical practice, both in terms of prognosis prediction and early diagnosis.

Moreover, another research investigated an innovative XAI framework to identify the key genetic biomarkers for a HCC prognosis [105]. In this study, the authors adopted robust AI models, validated against extensive gene expression datasets, and, by subsequently applying an XAI approach, demonstrated, at the same time, the predictive accuracy and the clinical relevance of the evidenced biomarkers (TOP3B, SSBP3, and COX7A2L) through explainable metrics [105]. Altogether, these results suggest that the application of XAI in biomarker discovery represents relevant progress in HCC research, offering a more complete understanding of the disease pathogenesis and concrete support to the currently available traditional diagnostic strategies.

Furthermore, a more recent study aimed to assess the efficacy of combining the automated ML (AutoML) with XAI in the identification of metabolomic biomarkers that can differentiate between HCC and liver cirrhosis in HCV-infected patients [106]. Particularly, the ML-TPOT tool was adopted to optimize the preparation of features and data, and the XAI-TreeSHAP approach was used to interpret the model by assessing the individual contribution of each metabolite to the categorization process. The authors reported a superior performance of the TPOT tool in distinguishing between HCC and cirrhosis compared to the other evaluated AutoML approaches, as well as the relative capability in identifying key metabolites, including L-valine, glycine, and DL-isoleucine, subsequently confirmed by the TreeSHAP analysis. TreeSHAP provided a comprehensive explanation of the contribution of these metabolites to the model’s predictions, thus increasing the interpretability of these findings [106]. 

To summarize, this evidence highlights that the combination of AI-based models with XAI has the potential to enhance the identification of biomarkers and to develop precise, easily interpretable, AI-driven solutions for diagnosing HCC.

In addition to the interpretation of AI-based-models-provided results, another relevant issue influencing the application of these tools in routine clinical practice is warranting the safety of the HCC patients and the relative data, constituting simultaneously a medical and an ethical question [108]. To objectivize the applications of trustworthy AI principles in research, the Assessment List for Trustworthy Artificial Intelligence (ALTAI) was recently proposed as a checklist (including seven items: human agency and oversight; technical robustness and safety; privacy and data governance; transparency; diversity, non-discrimination, and fairness; environmental and societal well-being; accountability) to help ensure that users can benefit from AI without being exposed to unnecessary risks by indicating a set of concrete steps for self-assessment [109].

Given this context, it is crucial to implement and diffuse trustworthy AI worldwide, by representing an approach to AI development that prioritizes safety and transparency for the people who interact with it; while ensuring its complete integration with XAI and traditional AI emerges as paramount when conceiving, designing, and validating all novel models aiming to improve the current management strategies of HCC [108,109].

Therefore, rather than imagining a context in which the clinician takes a backseat, the conscientious activity of new, highly trained figures appears to be essential to take full advantage of all the novel AI-dependent tools that will be developed in the future, without forgetting the main and noble aim of improving the HCC patient care.

In conclusion, now is not the time to update the diagnostic and predictive models’ armamentarium in HCC management. Although some important scientific battles have been won, the “war” against this human cancer is ongoing. In this scenario, the preconditions for a new era of precision medicine, where the “war efforts” of the scientific community, supported by the advantages offered by the application of AI to routine clinical practice, will lead to winning strategies, appear increasingly clear and give rise to hope for a significant improvement in the prognosis and quality of life of HCC-affected patients.

## Figures and Tables

**Table 1:** Principal AI-based models supporting US imaging in diagnosing HCC: main applications.

AI-Based Model	Description and Applications	Reference
DCNN-US	Deep convolutional neural network (DCNN) applicated to US imaging in differentiating benign from malignant liver lesions with an accuracy comparable to expert radiologists, CT-scan, and only slight inferior to MRI.	Yang et al. [63]
YOLOv5	DL techniques applicated to US imaging for detection and classification of seven different types of FLLs, including HCC and regenerative nodules.	Chaiteerakij et al. [64].
DCCA-MKL	A two-stage multiple-view learning framework for CEUS combining deep canonical correlation analysis and multiple kernel learning (DCCA-MKL), only adopting three typical CEUS images selected from the three phases of the exam (arterial, portal venous and late) to discriminates between benign and malignant FLLs.	Guo LH et al. [66]

HCC: hepatocellular carcinoma; DL: deep learning; FLLs: focal liver lesions; US: ultrasound; CEUS: contrast enhancement ultrasound; CT: computer tomography; MRI: magnetic resonance imaging.

**Table 2:** Principal AI-based models supporting CT and MRI imaging in diagnosing HCC.

AI-Based Model	Description and Applications	Performance and Accuracy	Reference
DeltaV-A_DWT1_LL_Variance-2D	Application of ML techniques on CT imaging features of cirrhotic patients for the training, calibrating, and validating a signature distinguishing HCC from non-HCC lesions with elevated accuracy by quantifying changes between arterial and portal venous phases	AUC: 0.740 (95%CI: 0.610–0.801)	Mokrane et al. [67]
ST3DCN	DL techniques applicated to CT imaging for excluding HCC, particularly targeting indeterminate observations	AUC: 0.919 (95%CI: 0.903–0.935) NPV: 0.966 (95% CI: 0.954–0.979)	Ho Yu et al. [68]
DCCA-MKL	DL algorithm created using CNN on MRI (enhanced and unenhanced) images and clinical data able to classify HCC with accuracy comparable to that of the experienced radiologists	AUC: 0.985; (95%CI: 0.960–1.000)	Zhen et al. [70]

HCC: hepatocellular carcinoma; DL: deep learning CT: computer tomography; MRI: magnetic resonance imaging; AUC: area under the receiver operating characteristic curve; CI: Confidence interval; NPV: Negative Predictive Value.

## Abbreviations

The following abbreviations are used in this manuscript:

HBLTs	hepatobiliary liver tumors
HCC	hepatocellular carcinoma
AF	advanced fibrosis
HBV	Hepatitis B virus
HCV	Hepatitis C virus
MASLD	Metabolic Dysfunction-Associated Steatotic Liver Disease
AI	artificial intelligence
ML	Machine learning
DL	Deep learning
MRI	magnetic resonance imaging
CAD	computer-aided detection
CADx	computer-aided diagnosis
TCGA	The Cancer Genome Atlas
DLCS	deep learning radio-clinical signature
LAGC	locally advanced gastric cancer
OS	overall survival
CP	Child-Pugh score
US	Ultrasound
EASL	European Association for the Study of the Liver
CT	computer tomography
NC-AMRI	non-contrast abbreviated magnetic resonance imaging
CEUS	contrast enhancement ultrasound
SVR	sustained virological response
RFS	random survival forest
AFP	alpha-fetoprotein
GGT	gamma-glutamyl transferase
BMI	body mass index
ALP	alkaline phosphatase
FFL	focal liver lesion
BCLC	Barcelona Clinic Liver Cancer
TACE	transarterial chemoembolization
TL	transfer learning
XAI	eXplainable Artificial Intelligence
AutoML	automated machine learning
